# Necroptosis and the RIPK1–RIPK3–MLKL pathway in chronic kidney disease: mechanisms, crosstalk, and therapeutic opportunities

**DOI:** 10.1080/0886022X.2026.2653414

**Published:** 2026-04-27

**Authors:** Jiawei Lu, Ying Wang, Yalin Cheng, Shuyuan Zhou, Yunqiang Liao, Jiqiang Zeng, Shanrong Zhang, Yang Tang

**Affiliations:** ^a^ First Clinical Medical College, Gannan Medical University, Ganzhou, Jiangxi, China; ^b^ Department of Nephrology, First Affiliated Hospital of Gannan Medical University, Ganzhou, Jiangxi, China; ^c^ Medical Technology School, Gannan Medical University, Ganzhou, Jiangxi, China; ^d^ Department of Traditional Chinese Medicine, First Affiliated Hospital of Gannan Medical University, Ganzhou, Jiangxi, China

**Keywords:** Necroptosis, chronic kidney disease, MLKL, RIPK1/RIPK3, therapeutic target

## Abstract

Chronic kidney disease (CKD) is a prevalent global health concern with a high worldwide prevalence rate. It is defined by a steady deterioration in renal function, which causes toxins and metabolic waste to build up and cause systemic problems and multi-organ failure. The development and progression of CKD are influenced by a number of pathogenic factors, such as diabetes mellitus, hypertension, glomerulonephritis, and exposure to nephrotoxic chemicals. However, the precise underlying mechanisms remain incompletely understood. Necroptosis, a well-studied form of regulated cell death, is primarily mediated by the receptor-interacting protein kinase 1 (RIPK1) and receptor-interacting protein kinase 3 (RIPK3) signaling complex. Understanding necroptosis provides new avenues for therapeutic modulation of cillnehronic kidney disease, linking regulated cell death to fibrosis and inflammation. RIPK3 expression is upregulated up to five-fold in experimental CKD models, underscoring its pathogenic significance. The definition, key molecular mechanisms, and most current advancements in pharmacological research pertaining to necroptosis are all comprehensively summarized in this article. This review provides mechanistic insights into necroptosis in CKD and highlights therapeutic targets for future translational research.

## GRAPHICAL ABSTRACT

**Figure:**
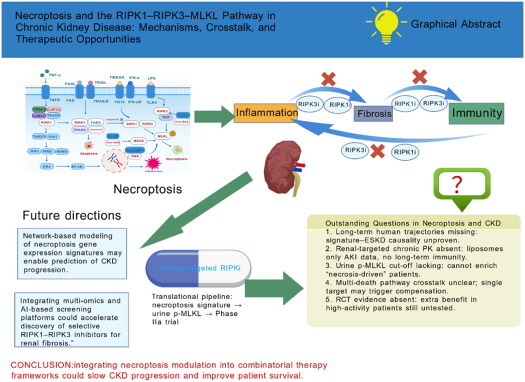

## 1. Introduction

CKD is the term for long-term structural and functional abnormalities of the kidney brought on by a number of different etiological factors. 13.4% is the stated prevalence worldwide [1], it is a non-communicable disease with high incidence and fatality rates. There is growing evidence that the main causes of the onset and progression of CKD include diabetes mellitus, arterial hypertension, glomerulonephritis, and exposure to nephrotoxic substances [2]. The mainstays of current therapeutic strategies are renal replacement therapy, dietary intervention, avoiding nephrotoxic drugs, and controlling modifiable risk factors. However, our understanding of the exact molecular processes controlling the development and course of CKD remains incomplete [3]. Necroptosis, an evolutionarily conserved modality of programmed necrosis, has emerged as a central driver of inflammation and fibrosis across multiple organs. Beyond the kidney, the RIPK1–RIPK3–MLKL axis fuels disease progression in liver fibrosis (e.g. nonalcoholic fatty liver disease), cardiac hypertrophy and heart failure, and neurodegenerative disorders such as Alzheimer’s disease and amyotrophic lateral sclerosis, positioning it as a trans-organ therapeutic target [4–7]. In nephrology, delineating whether this same pathway propels the inflammatory-fibrotic milieu of chronic kidney disease is now an urgent priority. There is presently no one universally effective method to completely prevent, halt, or reverse CKD, although comprehensive therapies can slow development, improve patient prognosis, and partially reduce the risk of disease onset [8]. Therefore, there is an urgent need for more research into the precise pathogenic mechanisms of CKD as well as the creation of novel therapeutic strategies [9]. Recent studies have markedly upregulated the crucial molecule RIPK3 in several CKD models. It has been demonstrated that the RIPK3-Mixed Lineage Kinase Domain-Like Protein (MLKL) signaling axis activates mitochondrial calmodulin-dependent protein kinase II and promotes renal cells’ synthesis of extracellular matrix as CKD progresses, suggesting that necroptosis plays a critical part in the pathogenesis of CKD [10,11]. Thus, it is of significant scientific and therapeutic importance to understand necroptotic pathways in CKD and their possible applications in the prevention and therapy of the disease.

**Figure 1.**
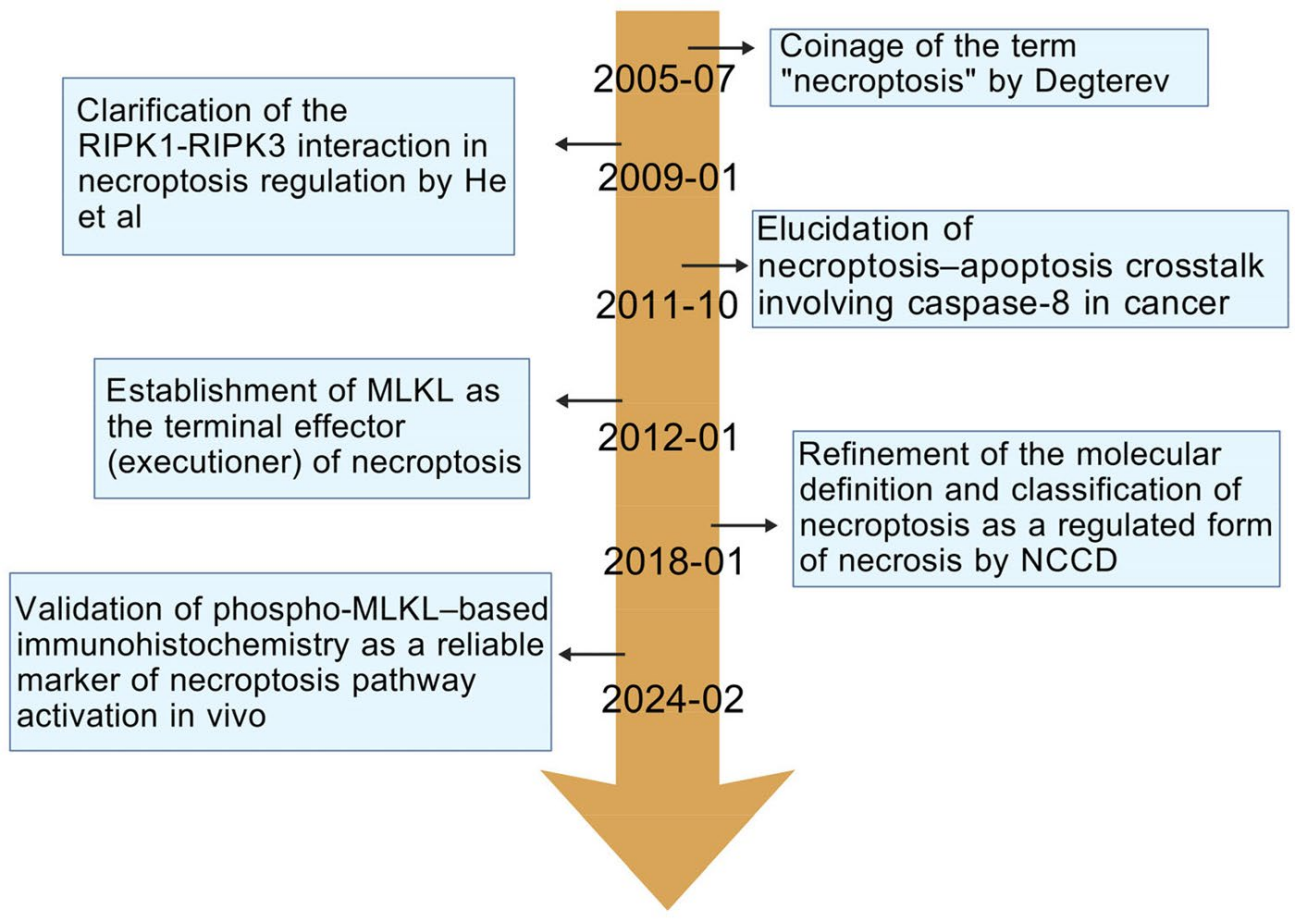
2005–2025 necroptosis research timeline.

Necrostatin-1 was found as a particular inhibitor of the process by Degterev et al. [12] in 2005, when they initially demonstrated that non-apoptotic programmed cell death can be caused by death receptor signaling, which they called “programmed necrosis” (also known as necroptosis) (Figure 1). The original name for necroptosis, a caspase-independent form of programmed cell death that exhibited traits of both necrosis and apoptosis was known as RIPK1-dependent necrosis. Necroptosis induces morphological changes, including organellar enlargement, nuclear and cytoplasmic disintegration, and rupture of the plasma membrane [13]. The roles of RIPK1 and RIPK3 in activating downstream effector molecules and their interaction in necroptosis control were further outlined by He et al. [14] in 2009. In 2012, Sun et al. [15] identified MLKL as the primary executor of necroptosis. Its phosphorylation and oligomerization are crucial processes that lead to increased membrane permeability and cellular death. The development of MLKL inhibitors like GW806742X and Necrosulfonamide (NSA) later supported the idea that MLKL plays a key role in necroptosis [14,16]. In addition to offering new therapeutic targets and approaches for the treatment of related diseases, these findings have significantly broadened the theoretical framework of controlled cell death [17]. The molecular processes of necroptosis will be the main emphasis of this review, which will also methodically look at how it contributes to fibrosis and chronic kidney disease, critically assess existing treatment modalities, and offer an outlook on how CKD treatment techniques may evolve in the future. Integrating multi-omics and AI-based screening platforms could accelerate discovery of selective RIPK1–RIPK3 inhibitors for renal fibrosis.

## 2. Cell death patterns and their multifaceted functions in CKD

**Figure 2.**
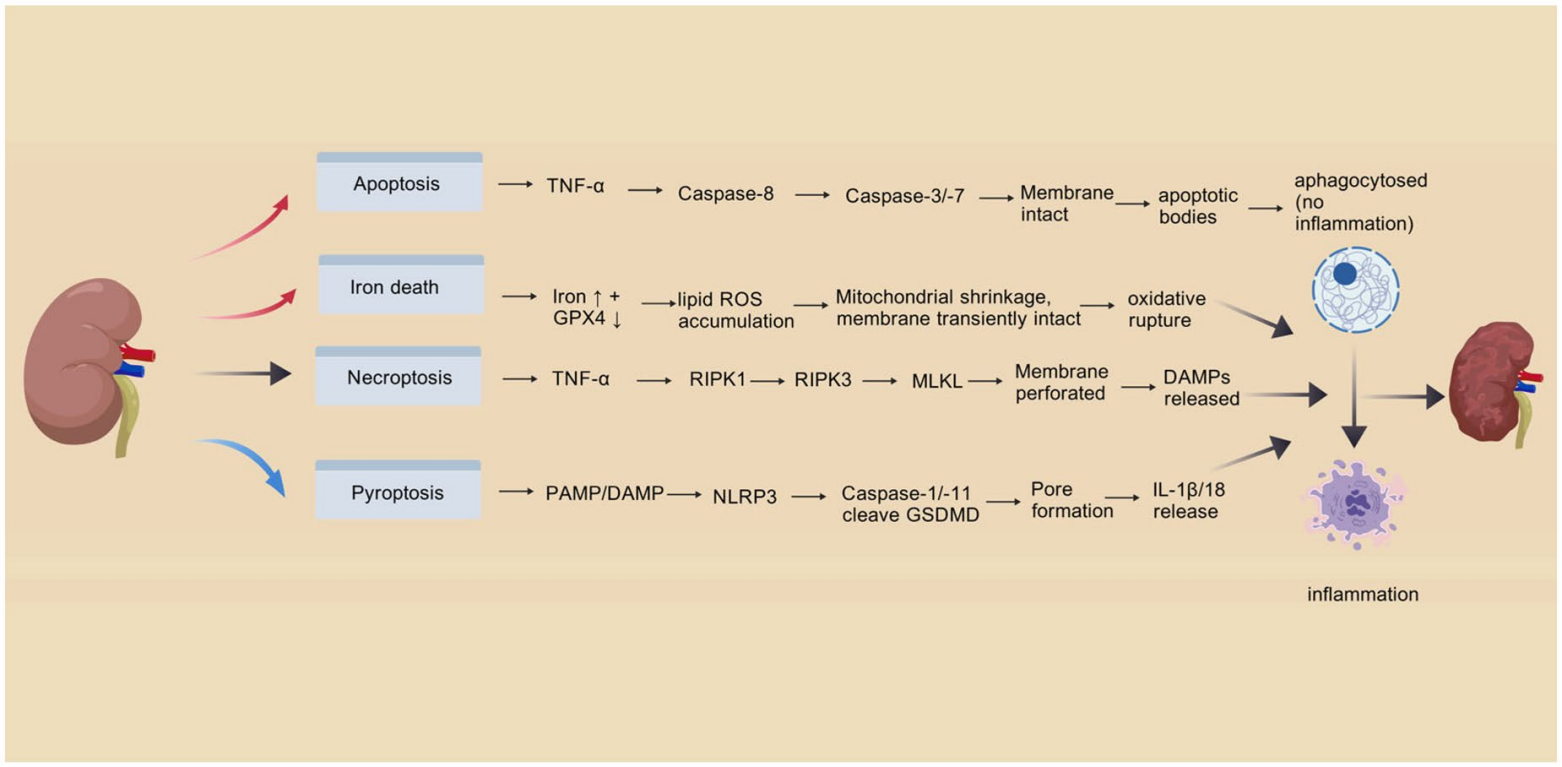
Different programmed cell death patterns in chronic kidney disease.

The pathophysiology of CKD implicates numerous cell death pathways (Figure 2). Despite their mechanistic differences, these modalities share activating stimuli and molecular interactions that ultimately lead to cellular death and functional deterioration [18]. Autophagy and programmed cell death—which includes apoptosis, pyroptosis, necroptosis, and ferroptosis—have an impact on the fate of renal cells (Table 1). Apoptotic death is the typical method of programmed cell death. Controlled necrosis, which is characterized by the rupture of the plasma membrane, the release of intracellular material, and the subsequent activation of inflammation, includes pyroptosis and necroptosis. In its early phases, ferroptosis—a form of cell death brought on by iron-dependent lipid peroxidation—has a relatively lower inflammatory response because the membrane integrity remains unchanged [18].

**Table 1.** Characteristics of different cell death types in chronic kidney disease.

Cell Death Type	Membrane Characteristics	Cytoplasmic Characteristics	Nuclear Characteristics	Notes	References
Apoptosis	Intact in the early stage, buds form apoptotic bodies in the late stage	Condensed, organelles relatively intact, forming apoptotic body cores	Chromatin condensed and marginalized, the nucleus fragmented into small bodies	Typical programmed cell death	[19]
Necroptosis	MLKL insertion forms pores, increases permeability, and eventually ruptures	Cellular swelling, organelles swell, and mitochondrial cristae rupture. Proinflammatory factors accumulate	Chromatin diffusely condensed, late-stage nuclear lysis	Regulated form of necrosis	[20].
Pyroptosis	Gasdermin forms pores, increases permeability, and eventually ruptures	Inflammasome assembly, formation of ASC specks, and pro-inflammatory factors accumulate	Intact in the early stage, secondary nuclear damage in the late stage, and no obvious chromatin condensation	Inflammatory programmed cell death	[21]
Ferroptosis	Membrane structure damaged by lipid peroxidation, blistering, increased permeability	Accumulation of lipid peroxidation products, mitochondrial damage, and iron ions is involved in reactions	Nuclear structure intact, no abnormal chromatin	Iron-dependent lipid peroxidation	[22]
Autophagy	Autophagosomes fuze with lysosomes, and the cell membrane remains intact	Increased autophagosomes and autolysosomes, degradation of damaged components (accumulation of damaged substances when function is defective)	Nuclear structure intact (possible nuclear condensation when autophagy is excessive)	Cell survival mechanism, but can lead to cell death under certain conditions	[23].

During the course of CKD, apoptosis is carried out by caspase cascade events in renal tubular epithelial cells *via* either the death receptor pathway or mitochondria. There is growing evidence that the key mechanisms behind renal tubular atrophy are cellular senescence and apoptosis in renal tubular epithelial cells [24]. Nuclear Factor-κB (NF-κB), p53/p21/p16, and the Wnt/β-catenin signaling pathway (Wnt/β-catenin) are a few of the signaling cascades that control apoptotic events [25].

Necroptosis, the archetypal regulated form of necrosis, has been recently described as necrotic cell death reliant on RIPK1 kinase activity. The activation and phosphorylation of RIPK3 by stimulation leads to the phosphorylation of MLKL, which ruptures the membrane and releases damage-associated molecular patterns (DAMPs) that disrupt the plasma membrane [26]. It has been demonstrated that renal tubular epithelial cell necrosis in CKD patients exacerbates renal interstitial fibrosis [20].

Ferroptosis in CKD may affect the metabolism of glutathione (GSH) and amino acids in renal cells [27]. A suppression of cysteine/glutamate transporter activity, which lowers intracellular GSH levels and increases oxidative stress, may trigger ferroptosis [22].

Pyroptosis relies on the cleavage of Gasdermin D by caspase-1/4/5/11 to create membrane holes and release pro-inflammatory mediators, including Interleukin-1 beta/18 (IL-1β/18) [28]. Recent studies have clarified the mechanisms underlying pyroptosis in murine CKD models, showing that hyperglycemic conditions can increase caspase-1 activation, upregulate the expression of IL-1β and IL-18, and cause pyroptosis in tubular epithelial and glomerular endothelial cells, all of which can hasten kidney damage. In CKD, pyroptosis inhibition reduces vascular calcification [21].

Cells use autophagy, a lysosome-dependent mechanism, to break down damaged or unnecessary organelles. It provides renoprotection in stressful situations and plays two roles in CKD [29]. In contrast, prolonged autophagy activation becomes harmful after severe injury, causing cellular senescence and promoting fibrosis *via* pro-fibrotic cytokine secretion [30]. Autophagy deficiency increases renal vulnerability to injury, leading to impaired function, damaged mitochondria accumulation, premature renal aging, and exacerbated fibrosis.

Research has shown that the regulation of cell death by apoptosis and necroptosis depends on intracellular signaling networks. Cells may die *via* necroptosis if caspase-8, a crucial apoptotic regulator, is blocked [31]. Necroptosis and autophagy also show reciprocal control. In contrast, necroptosis activation can hinder autophagy, such as when RIPK1 activation inhibits autophagy [32]. Autophagy can reduce necroptosis by removing damaged mitochondria and organelles, which lowers intracellular damage signals [33]. While modest Reactive Oxygen Species (ROS) can activate protective autophagy, chronic oxidative damage leads to excessive autophagy toward pro-death pathways [34,35]. Oxidative stress, through the rise of ROS, can cause apoptosis, necroptosis, ferroptosis, and pyroptosis [14,36–38]. It will be easier to precisely construct targeted intervention techniques if the cross-regulatory mechanisms of the molecular pathways driving different death modes are systematically analyzed.

## 3. Molecular mechanisms of necroptosis

**Figure 3.**
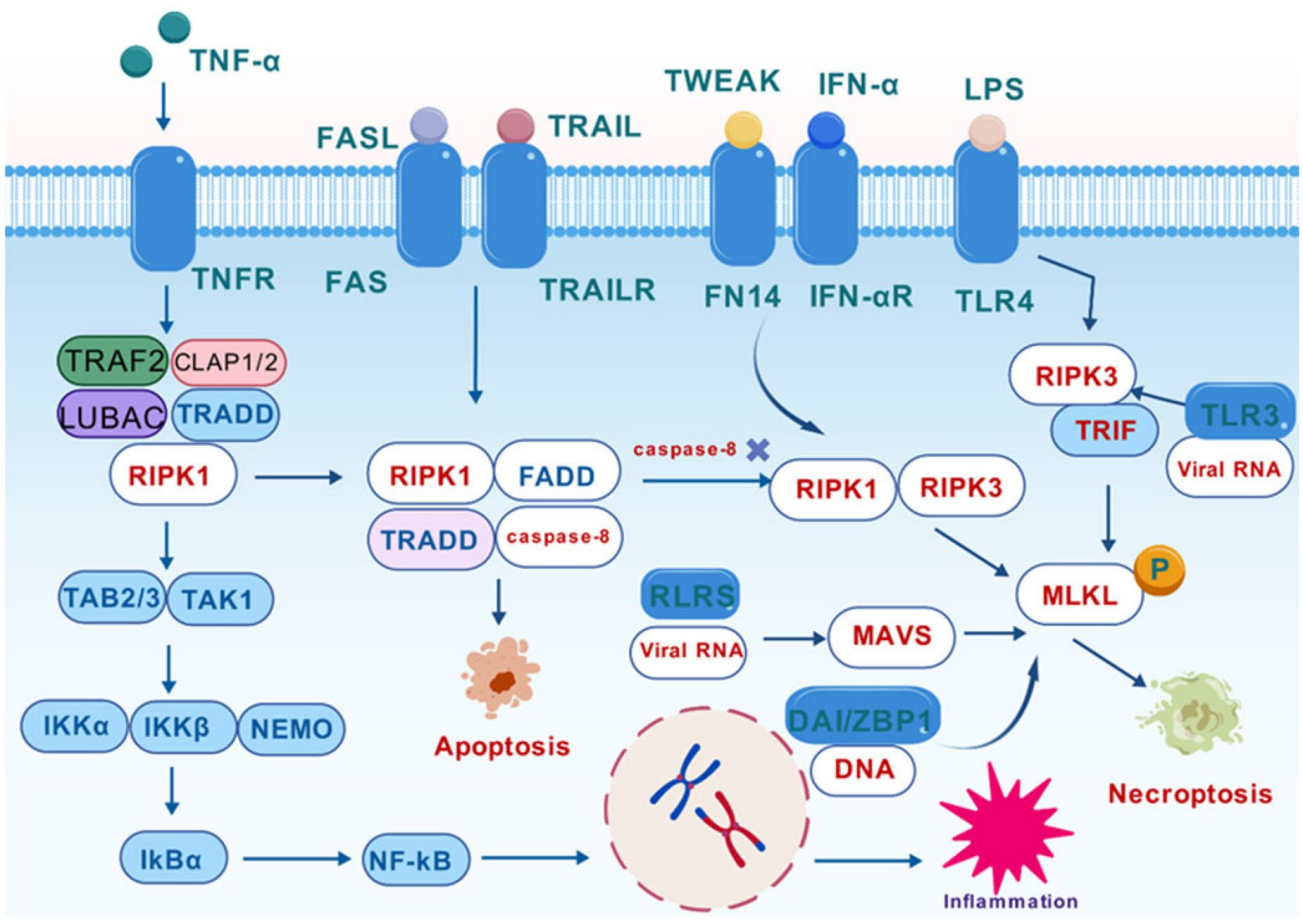
Necroptosis Molecular Mechanism.

Five classes comprise the necroptosis receptor network, which exhibits complementary and synergistic mechanisms: cytosolic DNA sensors DAI/ZBP1; pattern-recognition receptors Toll-Like Receptor (TLR)3/4 and RIG-I/MDA5 (RLRs); interferon receptors; Tumor Necrosis Factor (TNF)-like weak apoptosis inducer (TWEAK); and classical death receptors TNFR1, Fas/CD95, and TNF-related apoptosis-inducing ligand (TRAIL)-R (DR4/DR5) (Figure 3) [39]. Through the previously indicated ligand-receptor-dependent signaling pathways, necroptosis is triggered by extracellular stressors such as ischemia–reperfusion injury, Ca^2+^ overload, pharmacological insult, osmotic stress, and thermal stress [40]. The most researched pathway is the TNF-α pathway, which causes intracellular TNFR1- Death Domain (DD) trimerization by binding to cell-surface TNFR1 with high affinity, whether it is soluble or membrane-anchored. Through the DD, trimerized TNFR1 attracts TNF receptor-associated death domain protein (TRADD); in turn, TRADD attracts cellular inhibitors of apoptosis (cIAP1/2), TNF receptor-associated factor 2 (TRAF2), and linear ubiquitin chain assembly complex (LUBAC) to form complex I [41]. Complex I is stabilized by LUBAC-mediated linear ubiquitination of RIPK1, while complex I integrity is reinforced by cIAP1/2 catalyzing K63-linked ubiquitin chains [26]. The recruitment of TAB2/3-TGF-β-activated kinase 1 (TAK1) and the IKK complex (IKKα/IKKβ/NEMO) occurs; TAK1 activates the MAPK cascades (JNK, p38, ERK), inducing AP-1 transcription factors and amplifying inflammatory outputs, while IKK phosphorylates Inhibitor of κB α (IκBα), freeing NF-κB for nuclear translocation and transcription of pro-inflammatory/anti-apoptotic genes [42].

When ubiquitination is reversed by Cylindromatosis (CYLD) or Tumor Necrosis Factor alpha-induced protein 3 (A20) deubiquitinases, RIPK1 separates from complex I and moves into the cytosol. Complex IIa is formed by its association with TRADD and Fas-Associated Death Domain (FADD). RIPK1 recruits RIPK3 *via* the RIP Homotypic Interaction Motif (RHIM) to build complex IIb (necrosome) if caspase-8 activity is reduced; if caspase-8 activity is intact, caspase-8 is triggered and apoptosis occurs [43]. Through its kinase domain, activated RIPK3 phosphorylates the downstream substrate MLKL (Thr357/Ser358) [44]. Phosphorylated MLKL oligomerizes and translocates to the plasma membrane, where it binds membrane lipids like phosphatidylinositol phosphates (PIPs), causing permeabilizing pores or disrupting ionic homeostasis, causing cellular swelling, membrane rupture, and intracellular contents leakage. A “necrosis–inflammation” positive feedback loop is created when this process is accompanied by the production of DAMP (e.g. HMGB1, IL-1α), which activates nearby immune cells and intensifies the inflammatory cascade [44].

## 4. Key players in necroptosis and apoptosis regulation

### 4.1. Post-translational modification of RIPK1: cell survival and death are determined by “dual-track regulation” of phosphorylation and ubiquitination

**Figure 4.**
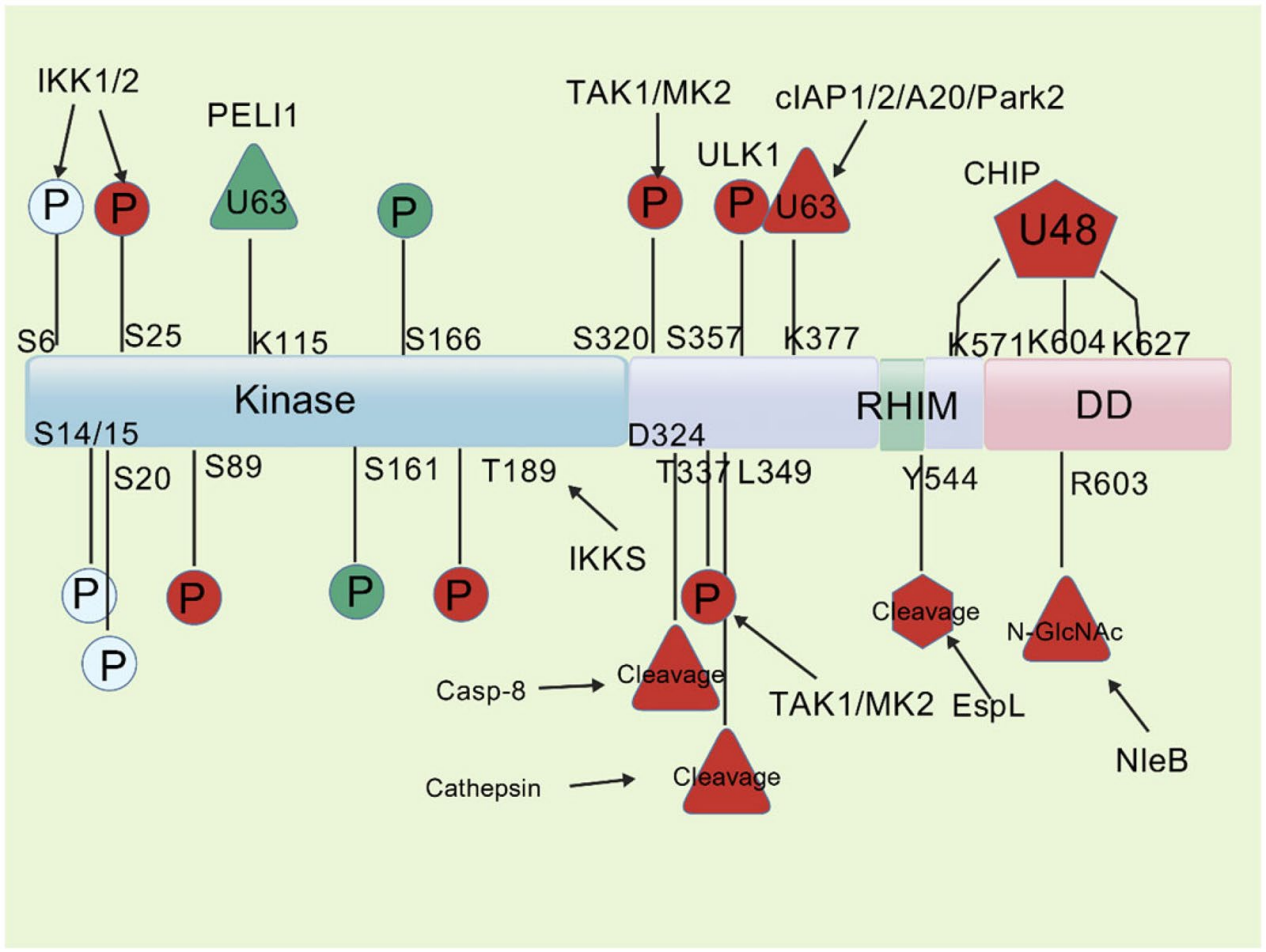
Post-translational modification landscape of RIPK1: phosphorylation, ubiquitination and cleavage events governing necroptosis signaling.

The fate of cells in terms of survival or death is influenced by a variety of factors, among which the dynamic balance of post-translational modifications of RIPK1 plays a crucial role, especially ubiquitination and phosphorylation that exert important regulatory functions (Figure 4) [45]. Under pro-survival conditions, E3 ubiquitin ligases (such as cIAP1/2 and LUBAC) mediate K63- or M1-linked ubiquitination of RIPK1, stabilizing complex I and activating the NF-κB signaling pathway [46]. In contrast, under pro-death conditions, deubiquitinating enzymes CYLD and A20 remove these ubiquitin chains, causing RIPK1 to translocate to the cytosol and form complex II [47].

A20 has two regulatory roles: it inhibits RIPK3 ubiquitination to prevent necrosome formation and changes K63-linked ubiquitin chains on RIPK1 to K48-linked chains, which encourages RIPK1 destruction by the proteasome [48]. Additionally, Spermatogenesis Associated 2 recruits CYLD to complex I to counteract LUBAC-mediated linear ubiquitination [49], while PELI1 catalyzes K63 ubiquitination of RIPK1 at K115, thereby enhancing the interaction between RIPK1 and RIPK3 [49].

The autophosphorylation of RIPK1 at S166 is one of the crucial processes in necrosome formation in terms of phosphorylation regulation, and its importance has been confirmed in a number of models of inflammatory diseases [50]. However, its role may vary depending on cell type and environment. TAK1-mediated phosphorylation of S14/S15 can synergistically enhance RIPK1 kinase activity, whereas inhibitory phosphorylation sites such as S25/T189/Y384 act as a “molecular brake” to limit RIPK1 kinase activity and maintain cellular homeostasis [51–53].

Disruption of the intricate regulatory network of RIPK1 may result in aberrant changes in cell death mechanisms and aid in the etiology of a number of diseases. However, the shift in cell death modes is the result of the interplay of multiple factors, and the disruption of the RIPK1 regulatory network is just one of the important factors involved.

### 4.2. The RIPK3-MLKL axis: necroptosis execution from membrane rupture to phosphorylation

**Figure 5.**
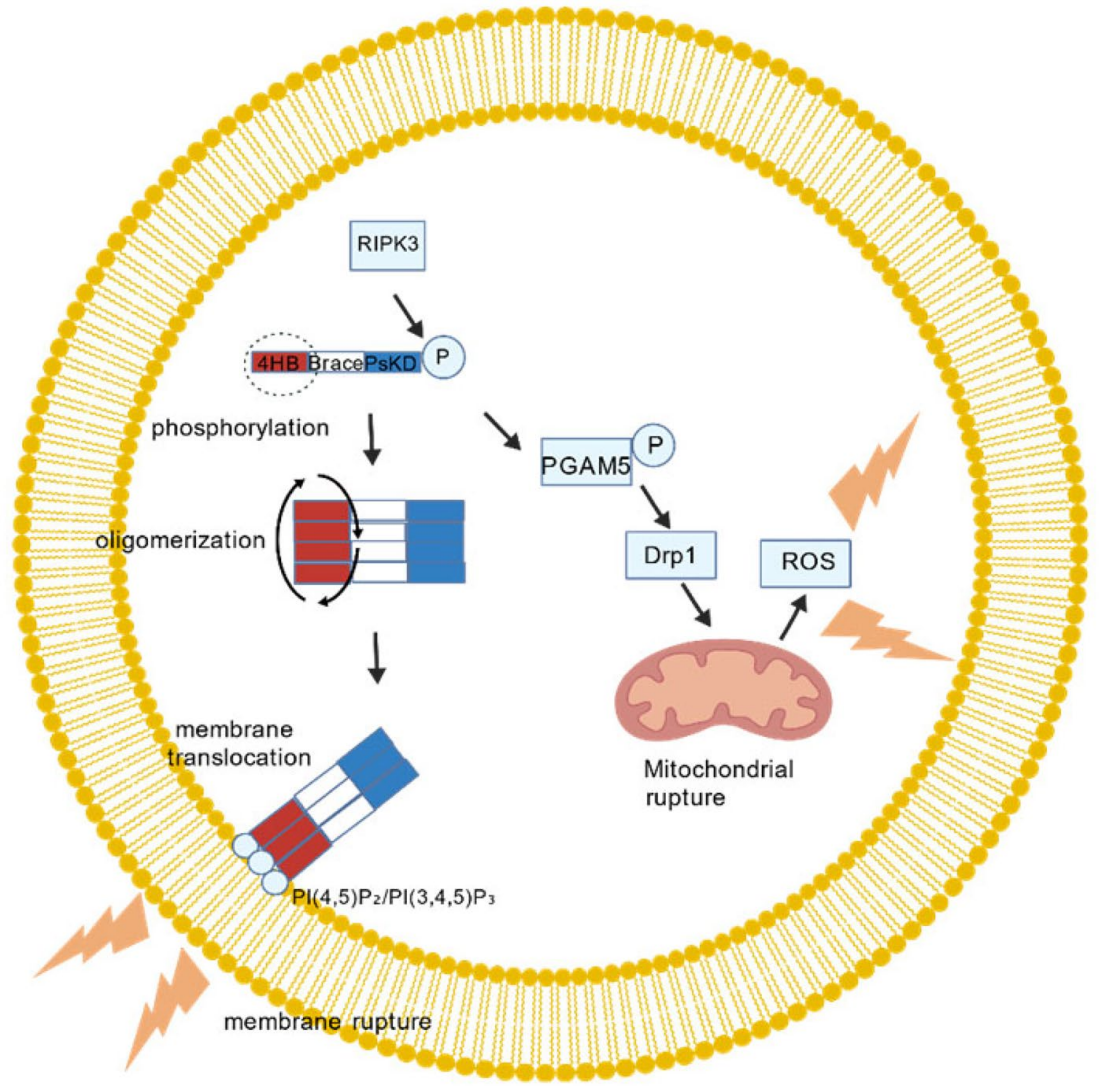
RIPK3-MLKL execution phase.

Through a “phosphorylation–oligomerization–membrane translocation–membrane rupture” cascade, the RIPK3–MLKL axis promotes necroptosis (Figure 5) [54]. The RIPK3 kinase domain phosphorylates key residues in the MLKL pseudokinase domain (human Thr357/Ser358, murine Ser345/Ser347). This reaction exposes the MLKL N-terminal four-helix bundle (4HB) and causes it to oligomerize into tetra- to octamers. After that, oligomerized MLKL passes through the 4HB into the inner leaflet of the plasma membrane, where it forms a nonselective cation channel and binds to PI(4,5)P_3_ and PI(3,4,5)P_3_ with high affinity. Concurrently, phosphorylated MLKL recruits PGAM5, which dephosphorylates and activates Drp1; excessive mitochondrial fission generates ROS bursts that feedback-inhibit Nrf2 nuclear translocation, thereby amplifying the necrotic signal and sustaining tubulointerstitial inflammation [55]. Thus, the RIPK3-MLKL-PGAM5-Drp1-ROS axis constitutes a self-amplifying mitochondrial-oxidative loop that links necroptosis to chronic renal injury. The process causes osmotic swelling, ionic imbalance, and membrane potential collapse, which ultimately leads to membrane rupture and cellular contents leakage [54]. The canonical TNF-α pathway still depends on RIPK1 to recruit RIPK3 through the RHIM domain [45], while TLR3/4-TRIF, ZBP1, and viral protein M45 (mouse cytomegalovirus M45) can directly activate RIPK3 *via* RHIM, highlighting RIPK3 as the direct executor of necroptosis [48,56,57]. Recent research has revealed that RIPK3 must first undergo oligomerization and autophosphorylation (Thr231/Ser232) before acquiring MLKL-phosphorylating capacity. Signaling hubs are RIPK1–RIPK3 connections mediated by RHIM; necroptosis is prevented by mutations or deletions in RHIM [58]. To bind RIPK3, TLR4-TRIF, ZBP1, and viral M45, use RHIM–RHIM competition [48,56,57]. TLR4-TRIF, ZBP1, and viral M45 form a complex signaling network through RHIM–RHIM competition that together alters the necroptosis start threshold and modality selection.

## 5. Necroptosis and chronic kidney disease

### 5.1. Necroptosis in diabetic kidney disease (DKD)

One important microvascular effect of diabetes, DKD affects 20–40% of people with the disease and is the primary cause of CKD and End-Stage Renal Disease (ESRD) [59]. Its basic pathophysiology can be summed up as “three highs–two processes–one sclerosis”: a persistently elevated blood sugar level triggers oxidative stress and the release of inflammatory factors like TGF-β and Vascular Endothelial Growth Factor, which in turn cause glomerular hyperfiltration, hyperperfusion, and hypertension through non-enzymatic glycation and polyol pathways (the three highs). The two processes then promote the synthesis of extracellular matrix while inhibiting its degradation, which leads to diffuse or nodular glomerulosclerosis with tubulointerstitial fibrosis (the one sclerosis). Clinically, the result shows up as increasing albuminuria and decreasing GFR, which eventually lead to renal failure [60]. Crucially, this cascade of “three highs, two processes, and one sclerosis” interacts with necroptotic signaling [61]. Death-receptor ligands like TNF-α cause necroptosis in hyperglycemic environments through TNFR1 and Fas; the most well-characterized kind of necroptosis is TNFR1-mediated. In response to TNF-α binding, TNFR1 forms necrosome complex IIb, pro-apoptotic complex IIa, or pro-survival complex I, which determines the fate of cells [62]. Necroptosis is linked to chronic inflammation and fibrosis in DKD because of recent findings showing that elevated glucose stimulates the RIPK1/RIPK3/MLKL axis in podocytes and tubular epithelial cells, causing mitochondrial breakage, ROS buildup, and inflammatory mediator release [32].

The first evidence that antioxidant intervention can prevent necroptosis was shown by Lin et al. [63] in 2018, who found that exogenous H_2_S inhibits the RIPK1/RIPK3/MLKL pathway, thereby reducing high-glucose-induced endothelial damage in human umbilical veins. Erekat et al. [64] discovered in 2022 that UCHL1 is a podocyte deubiquitinase that deubiquitinates RIPK1 and RIPK3, increasing necroptosis; overexpression of UCHL1 exacerbates podocyte necroptosis through the RIPK1/RIPK3 axis in high-glucose environments, leading to diabetic nephropathy. Qi Yu et al. [65] demonstrated in 2023 that p-MLKL levels are positively correlated with the severity of renal impairment, histological damage, and lipid-droplet accumulation in patients with DKD; in high-fat-diet db/db animals, the RIPK1 inhibitor RIPA-56 significantly reduced renal injury. The key mechanism was clarified in 2024: the RIPK1/RIPK3-MLKL-PGAM5-Drp1 axis is abnormally activated under high-glucose oxidative and inflammatory stress [66]. After RIPK3 phosphorylates MLKL, it recruits Phosphoglycerate Mutase Family Member 5 (PGAM5), leading to excessive mitochondrial fragmentation mediated by Dynamin-related protein 1 (Drp1), which damages the filtration barrier and the cytoskeletal architecture of podocytes [55]. In 2025, Zhimei Lv and associates [67] discovered that TRAIL/DR5 damages podocytes by inducing inflammation, apoptosis, and necroptosis, several forms of cell death. They divided DKD patients into two groups (C1/C2) according to their necroptosis characteristics; the C2 group has more immune cells (like CD8^+^ T cells and M1 macrophages), higher levels of inflammatory substances (like IL-1β and IL-18), and higher necroptosis markers (like p-MLKL), indicating that the interaction between necroptosis and the immune environment influences the different progression of DKD [68].

When taken as a whole, these results suggest that the RIPK1/RIPK3 cascade in DKD podocytes is a viable therapeutic target. DR5 inhibitors and UCHL1 deletion prevent necroptosis [67,69], while antioxidants decrease RIPK1/RIPK3 expression [63]. Guo et al. provided the first comprehensive *in vivo* evidence for necroptosis inhibitor therapy in DKD by demonstrating that Nec-1 significantly reduced urinary microalbumin, BUN, and ACR, attenuated glomerulosclerosis and interstitial fibrosis, and suppressed NF-κB-mediated inflammation in STZ/HFD diabetic mice [70]. Huangshuwai flowers, a traditional Chinese treatment, contained total flavonoids that reduced podocyte necroptosis in rats with DKD [71]. The RIPK3 inhibitor HS-1371 demonstrated selectivity and became the first small-molecule RIPK3 inhibitor to undergo preclinical evaluation in diabetic mice by increasing podocyte survival by 40% and reducing proteinuria by 60% without impacting apoptosis [72]. Through an RIPK3-dependent mechanism, curcumin has also been shown to stop podocyte damage brought on by high hyperglycemia [73]. Of note, botanical ingredients such as Abelmoschus manihot and curcumin have shown necroptosis-inhibiting potential in pre-clinical renal models. These findings remain confined to animal studies; their active moieties, pharmacokinetics, and human safety profiles have yet to be systematically characterized. They should therefore be regarded cautiously and positioned only as an exploratory adjunctive avenue until rigorous clinical validation is completed. These results suggest that necroptosis is a viable cellular target for the treatment of DKD. Nevertheless, the purported renoprotective benefits of RIPK1/RIPK3 inhibition remain unresolved. Necrostatin-1 has recently been shown to off-target indoleamine 2,3-dioxygenase, provoking immune-related adverse events [74]. Systemic RIPK3 ablation in mice causes lethal T-cell developmental defects within the first three postnatal weeks, underscoring systemic toxicity concerns [75]. Moreover, the overwhelming majority of current efficacy data have been derived exclusively from 8- to 12-week-old male rodents [76]. Consequently, future translational studies must mandate kidney-selective drug exposure—plasma concentrations below 20% of the IC_90_—coupled with immunotoxicity monitoring extending ≥12 months to rigorously balance therapeutic gain against potential risk.

### 5.2. Necroptosis in hypertensive nephropathy

Necroptosis plays a key role in hypertensive nephropathy (HTN-KD), a significant subtype of CKD caused by persistent hypertension that makes up 25–30% of all ESRD patients worldwide, much like it does in diabetic nephropathy [77]. Proteinuria→massive proteinuria→progressive GFR decline to ESRD is the clinical manifestation of its pathophysiology, which is characterized by the production of inflammatory mediators (such as TGF-β and IL-6), oxidative stress, renal hemodynamic disruption, and RAAS activation. These factors enhance ECM synthesis while suppressing degradation, ultimately leading to glomerulosclerosis and tubulointerstitial fibrosis [77]. Necroptotic signaling interacts with these processes [78].

Despite RAAS activation, Jiandong Zhang et al. [79] demonstrated in 2014 that TNF-α-deficient animals had attenuated blood-pressure responses and target-organ protection, suggesting renal parenchymal TNF-α involvement in the pathophysiology of hypertension. Eamonn Mehaffey et al. [80] showed in 2017 that TNF-α regulates renal hemodynamics through TNFR1 and TNFR2: activation of TNFR1 enhances natriuretic responses and prevents renal angiotensinogen generation, whereas activation of TNFR2 causes inflammation and renal damage. The inhibition of AT1R and AT2R considerably decreased tubular epithelial necroptosis in a model of renal damage produced by Ang-II, as seen by lower RIPK3 and MLKL expression, according to a 2019 study by Yongjun Zhu et al. [78]. They also identified Fas/FasL as downstream mediators of Ang-II-driven necroptosis.

All of these results point to necroptosis as a major cause of HTN-KD. Emerging therapeutic approaches target TNF-α and FasL: FasL suppression significantly reduces Ang-II-induced necroptosis in HK-2 cells [78], whereas TNFR1 activation may provide renoprotective blood-pressure regulation [80]. Necroptosis is therefore a new treatment option for HTN-KD.

### 5.3. Necroptosis in lupus nephritis and IgA nephropathy

Necroptosis’s pathogenic importance and mechanistic roles in IgA nephropathy (IgAN) and lupus nephritis (LN) have become major areas of study [81]. Because of the intricacy of the disease and the lack of effective treatments, necroptosis is a key factor in the pathophysiology of LN, a serious consequence and the primary cause of death in systemic lupus erythematosus (SLE) [82]. According to a 2020 study by Mingjiao Zhang et al. [83], necroptosis in LN was implicated by the correlation between renal involvement and disease activity and higher MLKL mRNA levels in peripheral blood mononuclear cells of SLE patients. In 2022, Xiao-Cui Chen et al. [84] showed that metformin decreased phosphorylated RIPK1 expression, enhanced renal function in MRL/lpr lupus-prone mice, and altered the AMPK/STAT3 pathway to prevent the formation of the RIPK1–RIPK3 complex while reducing inflammasome activation. A recent study in 2025 found that whole peony glycosides improve LN and inhibit ZBP1-mediated PANoptosis in podocytes, offering traditional Chinese medicine a new therapeutic justification [85]. Furthermore, Lin Peng et al. [75] found that RIPK1 activation caused mesangial cell necroptosis in MRL/lpr kidneys and that administering the RIPK1 inhibitor ZJU37 reduced mesangial cell necroptosis, which in turn relieved LN.

IgAN is mainly associated with mucosal immune dysregulation brought on by intestinal or upper respiratory infections, which results in abnormally glycosylated IgA1 that deposits immune complexes in the mesangium, activates complement, encourages the proliferation of mesangial cells and matrix expansion, and causes inflammatory damage [86]. IgAN research is still in its early stages; complement activation and oxidative stress brought on by galactose-deficient IgA1 (Gd-IgA1) deposition may trigger RIPK3 through TLR4 or TNFR, which would cause necroptosis of mesangial cells [87]. In renal biopsies from IgAN patients with crescent formation or capillary necrosis, clinicopathologic investigations showed markedly increased MLKL phosphorylation, which was associated with a deterioration in renal function [88]. To identify protein inhibitors that target IgAN-associated differentially expressed genes, recent research has used connection mapping, which may have therapeutic benefit for IgAN [89].

### 5.4. Necroptosis and renal fibrosis after renal ischemia-reperfusion injury

The common terminal pathway of almost all chronic progressive nephropathies is renal fibrosis [90]. Its hallmarks include controlled cell death and sterile inflammation, which eventually result in ESRD. Necroptosis’s role in ischemia-reperfusion injury (IRI) and the fibrogenesis that follows has garnered more attention recently [91,92]. Early studies found two apoptotic peaks during renal IRI, which corresponded to acute tubular injury and degenerative alterations throughout healing. Necroptosis also contributes to this condition, according to later research [61].

Seven days after UUO, Xia Xiao et al. [93] (2017) found that obstructed kidneys had higher levels of RIP1, RIP3, and MLKL protein and mRNA; Nec-1 treatment significantly reduced interstitial fibrosis, as seen by decreased production of TGF-β and α-SMA. Further linking necroptosis to fibrogenesis, Mitsuru Imamura et al. [94] showed in 2018 that RIPK3 induces fibrosis through AKT-dependent ATP citrate lyase activation. According to a 2020 study by Qin Dai et al. [95], fluphenazine reduced kidney fibrosis by blocking the RIPK3/MLKL necroptotic axis. This suggests that antifibrotic medications may also suppress necroptosis. In 2022, Shang Guo Piao et al. [96] identified a particular necroptotic mechanism in the advancement of fibrosis by revealing that RIP1–RIP3-mediated necroptosis causes fibrosis through the Wnt3α/β-catenin/GSK-3β cascade. According to a 2024 study by Mohamed A. Abou Taha et al. [97], sorafenib and edaravone, either separately or in combination, reduced fibrosis associated with obstructive nephropathy in rats by inhibiting the RIPK3/MLKL necroptotic pathway and reducing inflammation and oxidative stress. In 2025, a recent study confirmed necroptosis as a cause of fibrosis by showing that post-IRI reduction of RIPK1 or RIPK3 kinase activity reduced chronic kidney damage [98].

The significance of the RIPK1/RIPK3/MLKL axis in renal fibrogenesis is demonstrated by each of these findings. From the temporal peak of cell death following IRI to the activation of the RIPK3/MLKL pathway, pharmacological modulation, and the elucidation of specific signaling cascades, the study progressively clarified the intricate mechanisms by which necroptosis results in fibrosis. These investigations improve our knowledge of the renal fibrosis process and suggest possible therapeutic targets for the clinical management of the condition. methods for treating chronic renal disease that rely on necroptosis.

## 6. Important mediators that prevent necrotic death

### 6.1. Inhibitors of RIRK1

**Figure 6.**
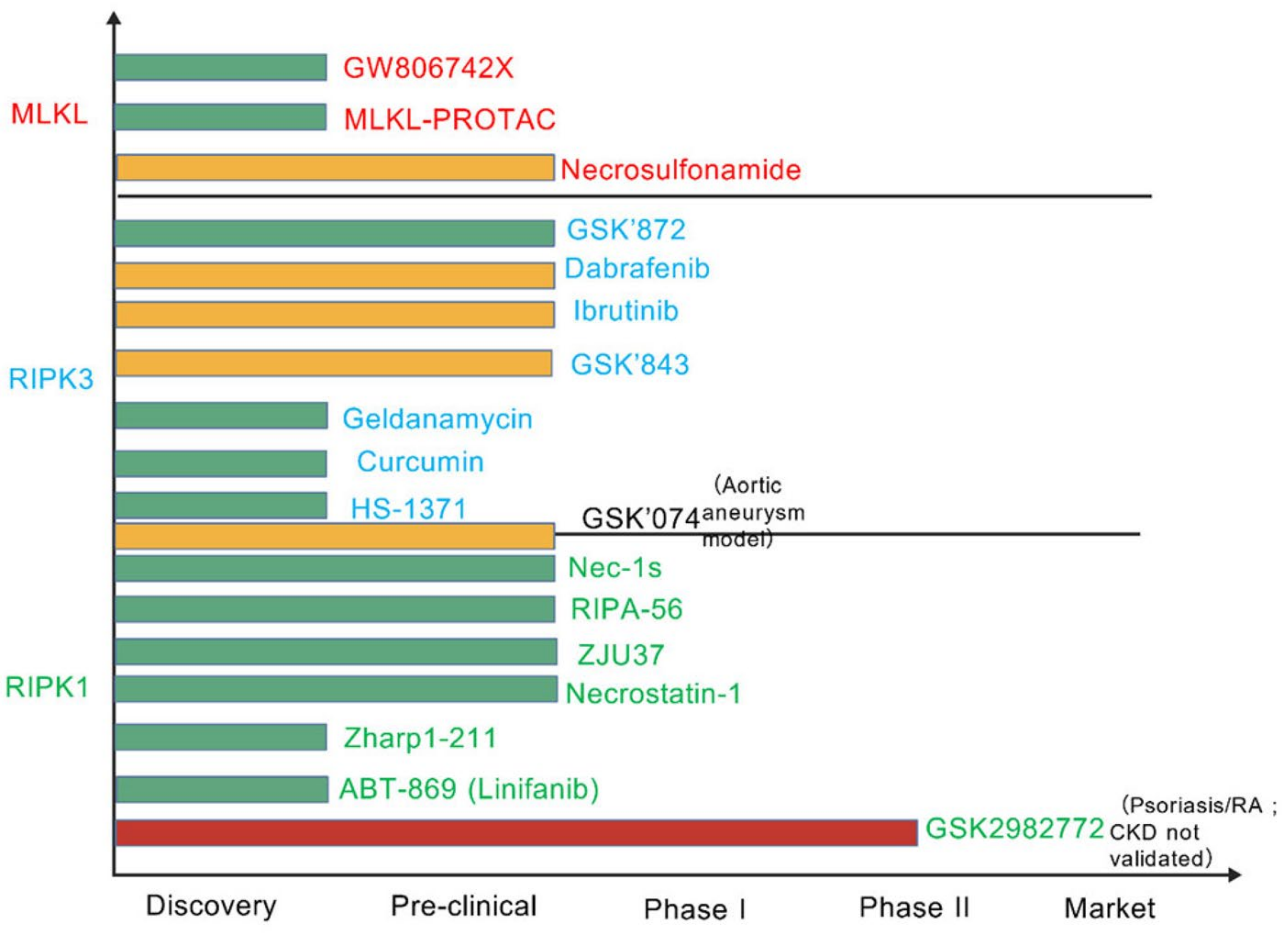
Treatment pipeline diagram (RIPK1, RIPK3, MLKL inhibitors).

A possible treatment approach for kidney diseases is the regulation of necroptosis, and investigating therapies that target this pathway has important clinical ramifications (Figure 6; Table 2). Researchers have assessed numerous strategies targeting the necroptotic axis, including both small-molecule inhibitors and gene engineering approaches [113]. The first inhibitor discovered, Necrostatin-1 (Nec-1), specifically inhibits RIPK1-mediated signaling and significantly reduces cell death in murine renal ischemia models (Table 3) [70]. Its successor, Nec-1s, has improved stability and selectivity, reducing downstream phosphorylation of MLKL and RIPK3 and inhibiting RIPK1 activity to decrease renal inflammation [114]. Other small-molecule inhibitors include GSK2982772, which showed favorable target engagement in Phase II trials [99], Yet a 2021 head-to-head study found GSK2982772 failed to outperform placebo once trough levels fell below IC_90_, highlighting the gulf between target engagement and clinical efficacy [116]; ABT-869 (Linifanib), discovered in 2023, which suppresses necroptosis in renal tissue [100]; RIPA-56, which demonstrated therapeutic potential in metabolic kidney disease by reducing necroinflammation and lipid accumulation in high-fat-diet db/db mice [65]; Zharp1-211, evaluated primarily in graft-versus-host disease (GVHD) and offering insights for preventing chronic allograft nephropathy [101]; and the newly identified ZJU37, which attenuates necroptosis in MRL/lpr lupus-prone mice and may be a therapeutic target for autoimmune nephritides [82]. In addition to small-molecule inhibitors, gene engineering has also been explored, with the RIPK1-D138N kinase-inactive mutant resisting TNF-induced necroptosis by eliminating kinase activity while maintaining scaffolding functions that sustain NF-κB signaling, thus maintaining renal cell survival and physiological homeostasis [117]. However, the long-term safety profile of Nec-1 is still unclear, and its potential impact on other cellular functions requires rigorous clinical validation. Overall, these findings suggest that targeting the necroptotic pathway holds promise for the treatment of kidney diseases, however, further research is needed to fully understand the therapeutic potential and safety profile of these interventions.

**Table 2.** Potential drugs for chronic kidney disease based on necroptosis.

DrugCategory	Drug Name	Research Model	Main findings	References
RIPK1 Inhibitors	Nec-1	Diabetic mice	The significant increase in the expression levels of p-RIPK1/RIPK1, p-RIPK3/RIPK3, and p-MLKL/MLKL in the kidneys of DKD mice suggests that necroptosis is involved in the progression of DKD. The RIPK1 inhibitor Nec-1 significantly inhibits the activation of the RIPK1/RIPK3/MLKL pathway and improves renal function.	[70]
	RIPA-56	Diabetic mice	Ripa-56 therapy also drastically reduced the activity of ripk1 kinase in mouse kidney tissue, decreased the amount of downstream p-mlkl, and effectively blocked the necroptosis pathway.	[65]
	GSK2982772	Phase II clinical trial	With the increase of gsk2982772 trough concentration, its efficacy on immune-mediated chronic inflammation may be improved	[99]
	ABT-869	Mouse and human cells	ABT-869 inhibits necroptosis in mouse and human cells.	[100]
	Zharp1-211	Intestinal epithelial cells	Zharp1-211 can selectively inhibit the activation of jak1/stat1 signaling pathway by ripk1 in IECs	[101]
	ZJU37	Mrl/lpr mice	Zju37 inhibits ripk1 to reduce necroptosis of mesangial cells	[82]
RIPK3 Inhibitors	GSK’872	Mice	Gsk’872 is a small molecule inhibitor that specifically inhibits the binding of ripk3 to its kinase domain and plays an important role in inhibiting necroptosis and ripk3 dependent inflammation	[102]
	GSK′843	Mouse Podocyte	The ability of GSK ′ 843 to regulate ROS and ripk3 overexpression is expected to prevent podocyte pathology by preventing its progression to necroptosis.	[103]
	GSK′074	Mouse fibroblasts	GSK’074 exhibits dual inhibitory effects on RIPK1 and RIPK3 in various cell types.	[104]
	Dabrafenib	Mouse	Dabrafenib may become a novel therapeutic strategy for patients with spinal cord injury by inhibiting necroptosis.	[105]
	Ibrutinib	Mouse	Ibrutinib inhibited TNF induced necroptosis by inhibiting autophosphorylation of ripk3 kinase, interaction of ripk3 and mlkl, and metastasis of necrosome.	[106]
	Geldanamycin	Neuronal cell	As an Hsp90 αinhibitor reduces the phosphorylation of mlkl.	[107]
	HS-1371	HT-29 cell	HS-1371 effectively restored the cell viability of TSZ mediated necrotic apoptosis in a dose-dependent manner, indicating that the novel small molecule HS-1371 acts as an RIP3 inhibitor.	[72]
	Curcumin	Mouse Podocyte	Curcumin, a powerful natural compound, has been shown to prevent podocyte damage caused by elevated glucose levels.	[103]
MLKL Inhibitors	NSA	Mouse	NSA can regulate necrotic apoptosis in MS mouse model.	[108]
	GW806742X	Mouse fibroblasts	*In vitro* studies on mouse fibroblasts have shown the ability to prevent necrotic apoptosis.	[109]
	necrosulfonamide	Rat	necrosulfonamide may confer protective effects in lung ischemia–reperfusion injury by suppressing necroptosis.	[110]
Other Potential Drugs	Melatonin	Swiss albino mice	Melatonin has successfully improved arsenic induced nephrotoxicity in both *in vitro* and *in vivo* models.	[111]
	TFA	Diabetic nephropathy rat model	TFA can improve necrotic apoptosis of DKD podocytes	[71]
	Metformin	Experimental ln mouse model	Metformin administration may inhibit RIPK1/RIPK3/MLKL mediated renal cell necrotic apoptosis through AMPK mediated STAT3 inhibition.	[84]
	Phytoestrogens	Chronic kidney disease models	CA pretreatment enhances the anti fibrotic activity of MSCs by inhibiting necrotic apoptosis induced by TGF - β 1/TNF - α/TNFR1 signaling.	[112]
	Sorafenib	Rat model of obstructive nephropathy	Sorafenib may block the RIPK-3/MLKL apoptotic pathway and inhibit renal oxidative stress and inflammation.	[97]
	Edaravone	Rat model of obstructive nephropathy	Edaravone may block the RIPK-3/MLKL apoptotic pathway and inhibit renal oxidative stress and inflammation.	[97]

**Table 3.** All interventions targeting necroptosis in kidney models.

Species	Renal Model (inducer)	Intervention	Route & Duration	Quantified Renal Outcomes	Key Limitations	Ref
Mouse	STZ/HFD diabetic kidney disease	Necrostatin-1	i.p. 1 mg kg⁻¹d⁻¹ × 8 w	↓ urinary micro-albumin 42 %, ↓ serum creatinine, ↓ glomerulosclerosis, ↓ cortical p-RIPK1/3/MLKL	Males only; no long-term toxicity	[70]
Mouse	db/db + high-fat diet	RIPA-56	p.o. 30 mg kg⁻¹ d⁻¹ × 12 w	↓ renal p-MLKL 46 %, ↓ lipid droplets, ↓ tubule injury score	Single dose; no female data	[65]
Mouse	Unilateral ureteral obstruction (UUO)	Necrostatin-1	i.p. 1.65 mg kg⁻¹ d⁻¹ × 7 d	↓ interstitial collagen 38 %, ↓ TGF-β/α-SMA, ↓ RIPK1/3/MLKL proteins	Acute obstruction; no metabolic CKD	[114]
Mouse	Ischemia-reperfusion (30 min clamp)	Whole-body RIPK3⁻/⁻	Genetic	↓ Day-1 serum creatinine 50 %, ↓ tubule necrosis, ↓ neutrophil influx	Systemic knock-out; T-cell developmental defects	[98]
Mouse	Ischemia-reperfusion	Whole-body MLKL⁻/⁻	Genetic	↓ tubule necrosis, ↓ DAMP release; double KO partially loses protection	Compensatory cell death unmasked	[115]
Mouse	Ang-II hypertension	Dual AT1R + AT2R blockade	oral × 4 w	↓ tubular RIPK3/MLKL proteins 35 %, ↓ Fas/FasL	Indirect inhibition; no direct necrosis inhibitor	[78]
Mouse	MRL/lpr lupus nephritis	ZJU37	oral 20 mg kg⁻¹ d⁻¹ × 6 w	↓ mesangial p-RIPK1, ↓ proteinuria 45 %, ↓ IgG deposition	Single strain; no chronic toxicity	[82]
Rat	Aristolochic acid CKD	Total flavones of *Abelmoschus manihot*	gavage 200 mg kg⁻¹ d⁻¹ × 8 w	↓ podocyte p-RIPK1/3, ↓ podocyte necrosis score	Active components undefined	[71]
Mouse	High glucose *in vitro*	HS-1371	0.5 µM × 24 h	↑ podocyte viability 40 %, ↓ p-RIPK3/MLKL	Cell level only; no PK data	[72]
Mouse	lupus nephritis	GSK872	i.p. 1.mg kg⁻¹ d⁻¹ × 14 d	↓ p-RIPK3/MLKL	Only female rats were used	[97]

### 6.2. Inhibitors of RIPK3

Selective RIPK3 kinase inhibitors, such as dabrafenib, GSK’872, and GSK’843, reduce RIPK3 activity by competitively occupying ATP binding sites; nevertheless, cytotoxicity may result from off-target kinase interactions [102,118]. In contrast, the dual-action inhibitor GSK’074 simultaneously targets RIPK1 and RIPK3, thereby disrupting necrosome formation. Pre-clinical murine models have demonstrated its low toxicity and high potency, positioning it as a promising complementary therapeutic strategy [104]. Ibrutinib directly targets RIPK3 to reduce TNF-induced necroptosis, according to recent data [106]. Moreover, HSP90/CDC37 complex inhibitors indirectly destabilize RIPK3; for instance, geldanamycin reduces RIPK3 expression by interfering with HSP90 chaperone function [107]. In cellular models, HS-1371 binds RIPK3 in an ATP-competitive manner, deactivating its catalytic site and stopping downstream signaling; its effectiveness in CKD has not yet been confirmed [72]. Curcumin, a recently discovered RIPK3 inhibitor, has the potential to mitigate hyperglycemic renal damage since it attenuates high-glucose-induced podocyte injury through a RIPK3-dependent mechanism [103]. It is worth noting that most of the aforementioned RIPK3 inhibitors exhibit ≥20% off-target kinase inhibition, and chronic systemic exposure can induce T-cell defects; therefore, kidney-targeted delivery remains the primary hurdle for clinical translation [119].

### 6.3. Inhibitors of MLKL

NSA and GW806742X disrupt MLKL oligomerization, delaying membrane translocation and preventing necroptotic signaling [108]. In mouse models of multiple sclerosis, NSA reduces necroptosis, while GW806742X inhibits it in mouse extracardiac fibers [109]. Other potential treatments include the MLKL-targeted necrosulfonamide [110]. A PROTAC molecule has recently been demonstrated to eliminate TSZ-induced necroptotic cell death and degrade MLKL [120]. While individual genetic deletion of RIPK3 or MLKL attenuates necroptosis and confers marked renoprotection, combined deficiency paradoxically diminishes this benefit [115]. A recent study reveals that RIPK3/MLKL-double-knockout mice exhibit a partial loss of renal protection, suggesting that the intricate interplay among cell-death modalities can unmask an alternative, previously unrecognized death pathway [115]. There is currently a lack of information regarding MLKL inhibitors in CKD, and specific CKD models are required to assess their mechanistic significance.

### 6.4. Targeting signaling molecules or upstream regulators implicated in necroptosis

Intervention against necroptosis can be accomplished by modifying upstream regulators or auxiliary signaling components in addition to directly targeting core necroptotic molecules like RIPK1 and RIPK3. For example, TNF signaling antagonists and Toll-like receptor (TLR) inhibitors prevent necroptotic cascades from starting [121], while improving negative regulators (A20) offers an alternate treatment option [122]. It is also beneficial to target the inflammatory environment that surrounds necroptosis; in renal disease, the self-amplifying loop between necroptosis and inflammation is broken by inhibiting important pro-inflammatory cytokines like interleukin-1β (IL-1β) or TNF-α [123]. Inhibitors that target upstream necroptotic checkpoints, such as TLR antagonists, TNF-α antagonists, and CYLD activators, are now the focus of research because they block upstream signaling, which reduces necroptotic initiation and the resulting tissue damage. Of them, TNF-α inhibitors have reached a somewhat mature state: etanercept and infliximab, which are already FDA-approved for the treatment of Crohn’s disease and rheumatoid arthritis, neutralize TNF-α activity, decrease cytokine production, and hence control necroptotic ignition [124]. However, possible side effects (such as an increased risk of infection) may limit the use of these biologics in CKD, requiring a thorough benefit-risk analysis. Another intriguing class consists of A20 activators, which are deubiquitinating enzymes that modulate RIPK1 and RIPK3 activity to negatively control necroptosis; increased A20 activity would increase its inhibitory effect on the necroptotic axis and reduce tissue damage. Animal studies have demonstrated that A20 can suppress disease progression by inhibiting necroptosis [122]. However, to date, no A20-specific activator has advanced to clinical trials, and its therapeutic potential in CKD requires further investigation.

### 6.5. Additional possible substances

Natural antioxidants are a class of natural compounds that can effectively prevent or delay oxidative reactions and are widely found in various natural sources, including plants, fruits, vegetables, spices, and herbs. In a mouse model of arsenic poisoning, studies have observed a significant elevation of necroptosis biomarkers. However, studies have shown that applying the natural antioxidant melatonin significantly protects the kidneys from arsenic toxicity. However, in-depth research still needs to further elucidate the specific mechanisms of action and optimal dosages of natural antioxidants in CKD [111].

Furthermore, in a rat model of DKD, TFA has been shown to significantly alleviate necroptosis in DKD podocytes by inhibiting the phosphorylation of RIPK1 and RIPK3 and lowering the expression levels of TNF-α and phosphorylated mixed lineage kinase domain-like protein (p-MLKL) in renal tissues [71]. Moreover, it has been established that Ang II-induced renal tubular cell necroptosis is a novel redundant mechanism that underlies Ang II-mediated renal tubular damage and chronic kidney disease. Studies have shown that blocking angiotensin II receptor 1 (AT1R) and angiotensin II receptor 2 (AT2R) can effectively alleviate Ang II-induced renal tubular cell necroptosis. Thus, inhibiting AT1R and AT2R could be a potential treatment strategy to lessen the excessive loss of renal tubular cells as CKD progresses [78].

Additionally, it has been shown that giving mice with experimental lupus nephritis (LN) metformin reduces renal injury, improves renal function, and increases the survival rate. The reduction of RIPK1/RIPK3/MLKL-mediated intrarenal cell necroptosis by metformin is thought to occur *via* AMP-activated protein kinase-mediated inhibition of Signal Transducer and Activator of Transcription 3 (STAT3). Nevertheless, patients with chronic kidney disease should use metformin cautiously since it may increase renal load, particularly in those with renal insufficiency [84].

By preventing necroptosis caused by the transforming growth factor-β1 (TGF-β1)/TNF-α/TNFR1 signaling pathway, phytoestrogen pretreatment has been demonstrated to significantly increase the antifibrotic activity of mesenchymal stem cells against chronic kidney disease. However, long-term use of phytoestrogens may pose hormone-related risks, such as endocrine disorders, so their application in chronic kidney disease should be carefully weighed against potential drawbacks [112].

In a unilateral ureteral obstruction animal model, it has been found that the combined administration of sorafenib and edaravone can significantly reduce renal injury in rats with obstructive nephropathy, possibly by blocking the RIPK3/MLKL necroptosis pathway and inhibiting renal oxidative stress and inflammatory responses [97].

## 7. From mechanistic insights to therapeutic translation

Necroptosis is not merely a mode of cell death but a central driver of the “fibrosis–inflammation–immunity” axis in CKD [125]. Upon activation of the RIPK1/RIPK3–MLKL cascade, plasma-membrane perforation releases DAMPs (HMGB1, IL-1α) that trigger sustained TLR4/MyD88/NF-κB signaling, amplifying renal inflammation. Concurrently, IL-6, TGF-β1 and PDGF synergistically activate fibroblasts, leading to collagen deposition and peritubular capillary rarefaction [126]. Finally, necroptotic debris recruits and polarizes CD8^+^ T cells and M1 macrophages, establishing an immune-positive feedback loop that stabilizes the fibrotic microenvironment. Consequently, kidney-restricted blockade of necroptosis simultaneously interrupts this triad axis and provides a mechanistic basis for reversing CKD structural remodeling [127].

For diagnosis and prognosis, p-MLKL staining intensity in renal biopsy or urinary p-MLKL levels represent promising noninvasive biomarkers of disease activity and fibrotic progression (Table 4) [128]. Therapeutically, combining RIPK1/3 inhibitors with standard-of-care SGLT2 inhibitors or RAS blockers may yield multi-target synergy to retard CKD progression [129]. As current anti-fibrotic therapy remains an unmet need in the 2024 KDIGO guideline, targeting necroptosis offers a novel mechanistic entry point [3].

**Table 4.** Clinical biomarkers of necroptosis in human CKD samples.

Biomarker (assay format)	Specimen	Disease cohort (n)	Primary renal endpoint	Key quantitative results*	Multivariate adjusted	References
**p-MLKL (Thr357/Ser358)** IHC H-score	Percutaneous renal biopsy	Diabetic kidney disease 62	Annual proteinuria rise & eGFR slope	H-score ≥ 3 vs < 3: proteinuria +2.1 vs +0.6 g/24 h/yr; eGFR −3.2 vs −0.9 mL/min/1.73 m²/yr	Yes (sex, HbA1c, baseline eGFR)	[32]
**RIPK3 mRNA** (qPCR)	Peripheral blood PBMC	Lupus nephritis 42 / non-renal SLE 46	Renal SLEDAI, 24-h proteinuria	LN fold-change ↑3.1; AUC 0.78 (95 % CI 0.68–0.88) for LN diagnosis; correlation with proteinuria *r* = 0.63	Yes (glucocorticoid dose)	[83]
**p-RIPK1(Ser166) + p-MLKL combined IF score**	Renal biopsy	IgA nephropathy 113	5-year ESRD risk	Score ≥ 3 high-risk: 5-yr ESRD 34 % vs 6 % low-risk; HR 4.85 (1.92–12.3)	Yes (Oxford MEST-C classification)	[88]
**UCH-L1 protein** (ELISA)	Urinary exosomes	Type 2 diabetic kidney disease 96	Annual eGFR decline	Top vs bottom quartile: eGFR −2.1 vs −0.8 mL/min/1.73 m²/yr; correlation with tissue p-MLKL *r* = 0.62	Yes (age, diabetes duration, baseline eGFR)	[64]
**RIPK3 protein** (Western blot)	Renal cortex	DKD 18, normal kidney 10	Semi-quantitative expression	5-fold up-regulation in CKD; correlation with interstitial fibrosis score *r* = 0.71	No multivariate model shown	[10]

Emerging kidney-targeted delivery systems for RIPK1 and MLKL inhibitors could redefine CKD therapeutic landscape by suppressing inflammation-driven fibrosis. CXCR4-ligand-modified pH-sensitive liposomes have increased renal targeting efficiency 8–30-fold while reducing plasma AUC by ∼60% in acute kidney injury models, providing a feasible nanocarrier platform for future RIPK1 inhibitors [130,131]. Similarly, miR-500a-3P-loaded liposomes directly inhibit RIPK3 and MLKL phosphorylation, suppress NF-κB activation, and attenuate cisplatin-induced AKI [132], establishing proof-of-concept for liposomal modulation of the necroptotic-inflammatory axis.

However, clinical trial readiness faces three structural hurdles.

1. Species divergence: Pre-clinical studies rely on rodent models that differ from humans in RIPK1/3 signaling, immune microenvironment, and drug metabolism. For example, the RIPK1 inhibitor SAR443060 exhibited neuroprotection in animals but compound-specific hepatotoxicity in long-term non-clinical toxicology studies that was not observed in short human trials, indicating incomplete predictive value [133].
2. Off-target effects: RIPK1/3 inhibitors may nonspecifically hit other kinases (e.g., RIPK2, IRAK4) or trigger scaffold-function-related adverse events. Certain RIPK3 inhibitors (e.g., GSK’872) can activate caspase-8-mediated apoptosis or disturb metabolic homeostasis (e.g., lipid metabolism), underscoring the need for improved selectivity [134].
3. Patient heterogeneity: RIPK1/3 pathway activity varies across diseases (Alzheimer’s, ALS, ulcerative colitis) and individuals. GSK2982772 failed in a Phase II ulcerative colitis trial, possibly due to disease-subtype, inflammatory background, or baseline RIPK1 expression differences. Moreover, genetic polymorphisms in RIPK1/3 may affect pharmacokinetics and target engagement, yet relevant biomarkers remain under-studied [116].

Key translational gaps include the absence of chronic CKD model data, lack of prospectively validated urinary p-MLKL/IL-18 cutoffs, and no human trials of kidney-targeted RIPK1 inhibitors.

Leveraging these pre-clinical findings, we propose a biomarker-enriched, first-in-human Phase IIa proof-of-concept trial: enroll CKD stages 3–4 patients with high exploratory urinary p-MLKL/IL-18 levels, randomize them to kidney-targeted RIPK1 inhibitor or placebo on top of standard RAS/SGLT2 blockade, and use 12-month eGFR slope as the primary endpoint to test whether nephron-restricted necroptosis inhibition slows inflammation-driven fibrosis.

With advances in single-cell transcriptomics and spatial proteomics, a “necroptosis signal atlas” can be constructed to identify RIPK1/3-high cell clusters (activated fibroblasts, injured podocytes) across CKD subtypes [135]. Integrating artificial intelligence models that fuze clinical features with molecular signatures will enable early progression-risk prediction. This positions necroptosis as an easy to handle precision medicine axis, explaining the heterogeneous clinical trajectory observed in CKD.

## 8. Prospects and challenges for the future

Future research on necroptosis in CKD should prioritize translating mechanistic insights into clinically actionable strategies by integrating kidney-targeted modulation of the RIPK1–RIPK3–MLKL axis with precision medicine frameworks. Key directions include (i) delineating cell type– and stage-specific roles of necroptosis across CKD etiologies using single-cell and spatial multi-omics to map a “necroptosis signal atlas”; (ii) developing kidney-restricted delivery systems and next-generation inhibitors with improved selectivity, pharmacokinetics, and long-term immunologic safety to overcome systemic toxicity; (iii) validating noninvasive biomarkers such as urinary or tissue p-MLKL and inflammatory mediators for patient stratification, prognosis, and pharmacodynamic monitoring; and (iv) designing biomarker-enriched, proof-of-concept clinical trials that combine necroptosis inhibition with standard-of-care therapies (e.g. RAS/SGLT2 blockade) to test effects on inflammation-driven fibrosis and eGFR decline. Coupling these efforts with AI-assisted drug discovery and predictive modeling will enable rational patient selection, optimize dosing strategies, and clarify crosstalk with other regulated cell-death pathways [136,137], thereby accelerating safe and effective translation of necroptosis-targeted interventions in CKD.

However, this therapeutic area also faces many challenges. Although RIPK3 inhibition is effective preclinically, its immunotoxicity is of concern; There is no feasible clinical protocol for renal targeted delivery strategy. The complex interactions between necroptosis and other cell death mechanisms (like apoptosis and pyroptosis) are still not fully understood, which makes it difficult to choose therapeutic targets and develop intervention strategies. Additionally, the precise mechanisms of necroptosis at various stages of chronic kidney disease are not fully understood, which restricts the application of precision medicine. There are also still few clinical studies assessing the effectiveness of necroptosis modulation in kidney diseases, and more extensive, long-term clinical trials are required to evaluate the safety, effectiveness, and long-term results of necroptosis-targeted therapies in clinical settings; Because of the intricate and varied pathophysiology of CKD, monotherapy that focuses only on necroptosis may not have the greatest therapeutic impact and must be used in conjunction with other forms of treatment.

Box 1. Key unresolved issues in CKD necroptosis research.

**Table:** 

Outstanding Questions in Necroptosis and CKD Long-term human trajectories missing: signature–ESKD causality unproven. Renal-targeted chronic PK absent: liposomes only AKI data, no long-term immunity. Urine p-MLKL cutoff lacking: cannot enrich “necrosis-driven” patients. Multi-death pathway crosstalk unclear; single target may trigger compensation. RCT evidence absent: extra benefit in high-activity patients still untested.

## 9. Conclusion

In summary, the RIPK1/RIPK3/MLKL complex orchestrates necroptosis, which is triggered by a broad receptor network and has become a key pathogenic driver in CKD. A thorough examination of its molecular makeup identifies a series of membrane ruptures and inflammatory amplifications that are common to autoimmune, hypertensive, and diabetic nephropathies. Heterogeneity in CKD pathogenesis and the complex interactions between necroptosis, apoptosis, pyroptosis, and autophagy indicate that patients require precision-targeted approaches. Current therapeutic paradigms, which range from direct inhibition of core necroptotic kinases to modulation of upstream receptors and downstream cytokines, have offered strong preclinical renoprotection. To convert these mechanistic insights into safe, effective treatments that will eventually stop the unstoppable course of CKD, rigorous clinical studies with validated biomarkers and prolonged follow-up are essential; integrating necroptosis modulation into combinatorial therapy frameworks could slow CKD progression and improve patient survival.

## Data Availability

Data sharing is not applicable to this article as no new data were created or analyzed in this study.
